# Molecular mechanisms of insect immune memory and pathogen transmission

**DOI:** 10.1371/journal.ppat.1010939

**Published:** 2022-12-15

**Authors:** Fabio M. Gomes, Melissa Silva, Alvaro Molina-Cruz, Carolina Barillas-Mury

**Affiliations:** 1 Laboratório de Ultraestrutura Celular Hertha Meyer, Instituto de Biofísica Carlos Chagas Filho, Universidade Federal do Rio de Janeiro, Rio de Janeiro, Brazil; 2 Laboratory of Malaria and Vector Research, National Institute of Allergy and Infectious Diseases, National Institutes of Health, Bethesda, Maryland, United States of America; Children’s Hospital of Philadelphia, UNITED STATES

## Introduction

Insect-borne diseases transmitted by mosquitoes, such as malaria, dengue, Zika, and lymphatic filariasis, remain among the most prevalent infectious diseases worldwide [1]. For example, the incidence of dengue infection has increased significantly in recent decades to more than 390 million cases per year, of which 96 million have clinical manifestations [2]. At the same time, the remarkable progress in malaria control programs has now staggered, and, in 2021, *Plasmodium falciparum* malaria incidence increased to more than 600,000 deaths [3]. While the deployment of insecticide-based strategies dramatically reduced the toll of insect-borne diseases in several regions, it resulted in widespread insecticide resistance in natural populations [4]. Thus, the development of new strategies to reduce disease transmission is greatly needed.

The immune response of an insect vector against a pathogen is a major determinant of vector competence, defined as the ability of a vector to transmit disease. Insect immunity is regulated by several different signaling pathways such as the JNK, JAK-STAT, Toll, IMD, and RNAi, which activate final effectors that limit pathogen development and replication [5,6]. Thus, immune priming and other mechanisms of immune memory that result in long-term enhancement of mosquito immunity have gained attention as important mechanisms to reduce disease transmission [7]. Here, we review recent discoveries on the molecular mechanisms mediating insect immune priming and its possible role in modulating the transmission of vector-borne diseases.

## Insects rely on an innate immune system that can activate a priming response

Like other invertebrates, insect defenses rely on their innate immune system, which shares some conserved features with that of vertebrates [8]. For decades, insects were thought to lack the ability to “learn” from previous exposure to pathogens because they do not have a classic adaptive immune system. However, this view has now been challenged by several studies demonstrating that insects can enhance their immune competence by activating a priming response. Immune priming has been defined as a functional state in which cells undergo long-lasting changes that enhance their response to a subsequent infection [9]. One of the first descriptions of innate immune priming in insects was in the cockroach *Periplaneta americana* [10] where the authors showed that immunization with killed *Pseudomonas aeruginosa* protected against infection with live bacteria. Similar events were later reported in several other insects (Table 1) [11–15], and in some cases, immune enhancement was shown to last for weeks [12]. For example, in *P*. *americana*, protection against *P*. *aeruginosa* was observed up to 14 days after priming [10], and a sublethal dose of *Streptococcus pneumoniae* also protected *Drosophila* flies for 14 days [13]. In these systems, however, it was not possible to demonstrate whether the observed protection was due to a long-lasting initial immune response or to the ability to mount a stronger immune response to the second challenge.

**Table 1:** Previous reports of insect immune memory have identified evidence for specific, nonspecific, transgenerational, and long-term immune memory depending on the insect and pathogen model.

Reference	Invertebrate Species	Pathogen	Specific Memory	Nonspecific Memory	Transgenerational Memory	Memory Across Life Stages
[27]	*Aedes aegypti*	*DENV*	Yes	No	No	Yes
[24]	*Aedes aegypti*	*Escherichia coli*	Yes	No	No	Yes
[70]	*Anabrus simplex*	*Metarhizium acridum*	Yes	No	No	No
[71]	*Anopheles albimanus*	*Plasmodium berghei*	No	Yes	No	No
[26]	*Anopheles gambiae*	*Escherichia coli*	Yes	No	No	No
[37]	*Anopheles gambiae*	*Plasmodium berghei*	No	Yes	No	No
[72]	*Anopheles gambiae*	*Escherichia coli*, *Enterobacter sp*. and *Staphylococcus aureus*	Yes	Yes	No	Yes
[31]	*Apis mellifera*	*Paenibacillus larvae*	Yes	No	Yes	No
[15]	*Bombus terrestris*	*Pseudomonas fluorescens*, *Paenibacillus alvei* and *Paenibacillus larvae*	Yes	No	No	No
[14]	*Bombus terrestris*	*Crithidia bombi*	No	Yes	Yes	No
[14]	*Bombus terrestris*	*Arthrobacter globiformis*	Yes	No	Yes	No
[73]	*Bombus terrestris*	*Arthrobacter globiformis*	No	Yes	Yes	No
[18]	*Bombyx mori*	*Photorhabdus luminescens*	Yes	No	No	No
[74]	*Caenorhabditis elegans*	*Pseudomonas aeruginosa*	Yes	No	No	No
[28]	*Daphnia magna*	*Pasteuria ramosa*	Yes	No	Yes	No
[13]	*Drosophila melanogaster*	*Streptococcus pneumoniae*	Yes	No	No	No
[16]	*Drosophila melanogaster*	Sindbis virus	Yes	No	No	No
[35]	*Drosophila melanogaster*	*Drosophila* C virus	Yes	No	No	Yes
[32]	*Drosophila melanogaster*, *Aedes aegypti*	Sindbis virus, *Drosophila* C virus, cricket paralysis virus, flock house virus	Yes	No	Yes	No
[11]	*Galleria mellonella*	*Photorhabdus luminescens*	Yes	No	No	No
[20]	*Galleria mellonella*	*Photorhabdus luminescens*	No	Yes	No	No
[25]	*Gryllus campestris*	*Serratia marcescens* (LPS)	Yes	No	No	Yes
[23]	*Haliotis diversicolor*	*Vibrio harveyi*	Yes	No	No	No
[75]	*Litopenaeus vannamei*	*Vibrio alginolyticus and Vibrio harveyi*	Yes	Yes	No	No
[76]	*Litopenaeus vannamei*	*Bacillus subtilis*	No	Yes	No	No
[21]	*Manduca sexta*	*Escherichia coli*	No	Yes	No	No
[77]	*Manduca sexta*	*Micrococcus luteus*	Yes	No	Yes	No
[10]	*Periplaneta americana*	*Pseudomonas aeruginosa*	Yes	Yes	No	No
[30]	*Plodia interpunctella*	*Plodia interpunctella granulosis virus*	Yes	No	Yes	No
[78]	*Drosophila melanogaster*	*Pseudomonas aeruginosa*	Yes	No	No	No
[12]	*Tenebrio molitor*	LPS	No	Yes	No	No
[79]	*Tenebrio molitor*	*Staphylococcus aureus*, *Bacillus thuringiensis*, *Escherichia coli* and *Serratia entomophila*	Yes	No	Yes	No
[80]	*Tenebrio molitor*	LPS (*Escherichia coli*)	Yes	No	Yes	No
[81]	*Tenebrio molitor*	*Arthrobacter globiformis*, *Bacillus subtilis*, *Escherichia coli* and *Serratia entomophila*	No	Yes	Yes	No
[29]	*Tenebrio molitor*	*Escherichia coli* (LPS)	Yes	No	Yes	No
[17]	*Tribolium castaneum*	*Bacillus thuringiensis*	Yes	No	No	No
[82]	*Tribolium castaneum*	*Bacillus thuringiensis* and *Escherichia coli*	Yes	Yes	Yes	No
[19]	*Tribolium castaneum*	*Escherichia coli*, *Bacillus thuringiensis thuringiensis* and *Bacillus subtilis*	Yes	No	No	No
[83]	*Tribolium castaneum*	*Bacillus thuringiensis*	No	Yes	Yes	Yes
[84]	*Tribolium castaneum*.	*Bacillus thuringiensis*	Yes	No	No	No
[85]	*Tribolium confusum*	*Gregarina minuta*	Yes	No	No	Yes

Depending on the model, the effect of priming can be pathogen specific [13,15–19] or nonspecific [20–23]. In *P*. *americana*, the long-lasting (14 days) protection against *P*. *aeruginosa* after priming with killed *P*. *aeruginosa* was reduced to 3 days when insects were challenged with a different bacterial species [10]. Priming can also extend across different life stages. For example, priming larvae can enhance adult immunity [24–27], and even transgenerational immune priming (TGIP) has been described in several models [28–32]. Interestingly, stress such as that inflicted by tissue injury [22], larval competition [33], or nutritional restriction [34] can also result in priming-like phenotypes, suggesting that understanding the mechanisms of these responses might provide further insights into the molecular regulators of immune priming. In summary, a plethora of evidence (Table 1) has confirmed that insect immunity shares memory-like components. However, the molecular mechanisms and immune pathways underlying these responses have only been established in a very limited number of model systems.

## Antiviral immune priming in *Drosophila*

*Drosophila* hemocytes enhance antiviral immunity in adult flies by taking up dsRNA and generating viral DNA (vDNA) that serves as a template to synthesize secondary viral siRNAs (vsRNA), which are delivered to other tissues by exosome-like vesicles [16]. Oral infection of fly larvae with *Drosophila* C virus (DCV) enhanced survival to a lethal challenge with the same virus as adults, although there was no difference in viral load, suggesting that previous exposure to the virus enhanced tolerance to infection in adult flies [35]. A strong TGIP was triggered when adult female flies were infected with positive single-strand RNA viruses. This enhanced antiviral immunity was passed to the offspring for up to five generations in a sequence-specific and RNA-dependent manner. Interestingly, TGIP was not mediated by the RNAi pathway [32]. Strong TGIP was also documented in the progeny of *Aedes aegypti* female mosquitoes infected with the chikungunya virus [32]. vDNA was suggested to be an important component of the antiviral immune memory, as it has been isolated in adult flies infected during larval stages [35] and in the progeny of infected adult females [32]. However, the mechanism of vDNA transfer and amplification and the cells involved remain to be determined.

## *Plasmodium* infection primes the *Anopheles* immune system

*Plasmodium*-induced enhanced immunity in *Anopheles* is a well-characterized model that provides some insights into the mechanism of insect priming. Briefly, *Plasmodium* infection results in a hemocyte-dependent state of enhanced immunity to subsequent infections [36]. Early work established that an increase in the proportion of circulating granulocytes—dynamic phagocytic hemocytes—mediates antiplasmodial immune memory [37]. This response is permanent, and the gut microbiota is required both to establish and recall the priming response, demonstrating that it is not due to a long-lasting immune response to the initial infection [37]. Later studies revealed remarkable coordination of insect immunity involving several different tissues and cell types, with eicosanoid lipids (prostaglandins and lipoxins) as key systemic signaling molecules.

**Fig 1:**
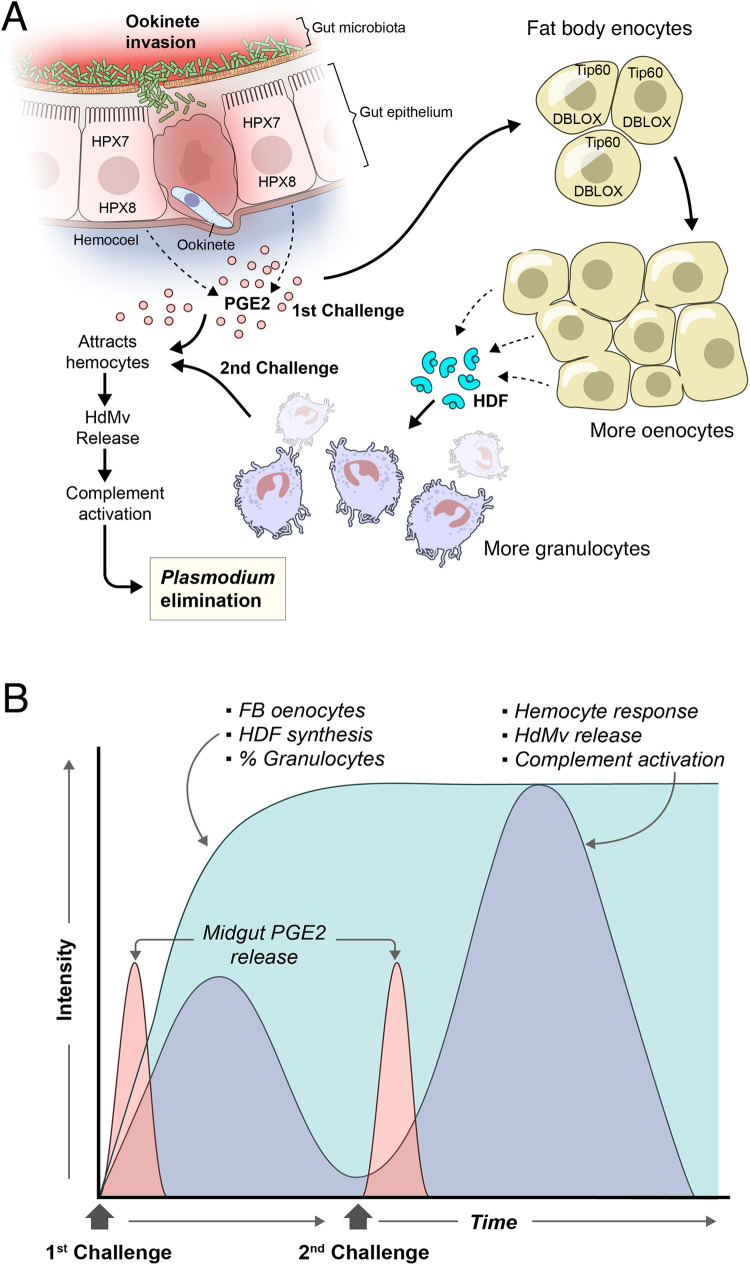
Immune priming in *An*. *gambiae* mosquitoes. (**A**) *Plasmodium* infection induces the expression HPX7 and HPX8 that mediate PGE_2_ synthesis by the midgut following microbiota contact with epithelial cells during ookinete invasion. The release of PGE_2_ triggers the production of the HDF by DBLOX-positive fat body oenocytes that proliferate following a Tip60-dependent mechanism. At the hemolymph, HDF induces the proliferation of circulating granulocytes, which are attracted to the midgut during reinfection following the PGE_2_ signal. Granulocyte release microvesicles (HdMv) at the site of recruitment, which mediates complement-like activation and *Plasmodium* elimination. Thus, the intensity of the mosquito immune response to *Plasmodium* can be enhanced by a previous infection. (**B**) Upon PGE2-dependent priming, the production of HDF in response to ookinete midgut invasion is constitutively enhanced following the first challenge, and this induces a constitutive increase in the proportion of circulating granulocytes. After the initial challenge, hemocyte association with the mosquito midgut goes back to basal levels. However, reprogramming of hemocytes during this first exposure results in enhanced hemocyte recruitment and a stronger immune response to a subsequent infection [37–40,43]. DBLOX, double-peroxidase; FB, fat body; HDF, hemocyte differentiation factor; HdMv, hemocyte-derived microvesicle; PGE2, prostaglandin E2.

*Plasmodium* infection induces the expression of two heme-peroxidases (HPX)—HPX7 and HPX8—which mediate prostaglandin E2 (PGE2) synthesis by the midgut, when the microbiota comes in contact with midgut cells during ookinete midgut invasion (Fig 1A) [38]. This systemic PGE2 release triggers the production of a hemocyte differentiation factor (HDF), that promotes hemocyte differentiation into granulocytes. HDF is a complex of lipoxin A4 (LXA4) bound to Evokin, a lipid carrier of the lipocalin family [39]. More recently, LXA4 synthesis was shown to require the activity of a third HPX—double-peroxidase (DBLOX)—produced by oenocytes, a subpopulation of fat body cells that proliferates in primed mosquitoes (Fig 1A) [40]. DBLOX and Evokin expression remain high after infection. The observation that a single systemic injection of PGE2 also triggers a long-lasting increase in DBLOX expression, and HDF release, suggested that epigenetic factors could be important mediators of immune priming. A functional screening in which all mosquito histone acetyltransferase (HATs) were silenced revealed that the HAT Tip60 is, indeed, essential for priming [40].

Ookinete invasion causes irreversible damage and invaded cells activate a strong nitration response as they undergo apoptosis [41,42]. Mosquito hemocytes are attracted to the basal surface of the midgut by PGE2, and undergo apoptosis when they come in contact with a nitrated surface, releasing microvesicles that promote mosquito complement-mediated elimination of ookinetes [43]. A mosquito hemocyte atlas was recently established using single-cell transcriptomics, and it identified new subpopulations of granulocytes that express specific markers [44]. Studies are underway to define the role of different hemocyte subpopulations in antiplasmodial responses and how priming affects hemocyte differentiation.

## Trained immunity as a memory feature of innate immunity

Recently, the concept of immune training (or trained immunity) has emerged as a key component of vertebrate innate immunity [45]. Like immune priming, trained immunity enhances the immune response to a second challenge. In trained immunity, transcription of immune effectors returns to a basal state after the primary challenge, but the final effector cells respond better to subsequent infections [9].

Immune training of monocytes by fungi infection comprises one of the best-studied models of trained immunity. Briefly, the presentation of fungal β-glucans induces an epigenetic reprogramming that increases cytokine release in response to a second exposure [46]. Similar to immune priming models, immune training lacks specificity, as exposure to fungal molecules also results in a protection against bacterial infections [47]. Similarly, bacille Calmette-Guérin (BCG) vaccines offer nonspecific protection against several different infections [48], and a role of BCG vaccine-induced trained immunity against SARS-COV-2 has been proposed [49]. At the molecular level, trained immunity is characterized by a shift in energy metabolism, including an increased rate of glycolysis. This metabolic shift is dependent on the mevalonate-induced TOR-HIF1α pathway [47,50]. Accordingly, inhibition of the TOR pathway during in vivo *Candida* mice infection prevented immune training and resulted in increased mortality during a subsequent immune challenge [47].

While the distinctive features of trained immunity are yet to be better described in insect models, insects also undergo some metabolic shifts following infection, like those in immune-trained vertebrate cells. For example, *Drosophila* macrophages switch to aerobic glycolysis when mounting an antibacterial defense [51]. Furthermore, this metabolic switch is also HIF1α dependent and is required for the survival of infected flies [51]. Interestingly, while HDF production and the proportion of circulating granulocytes remain constitutively elevated following an initial *Plasmodium* infection (Fig 1B), the ability of hemocytes to mount a more effective response to a second *Plasmodium* challenge is dependent on the presence of the bacterial gut microbiota both at the time when priming response is established and to elicit a stronger response to the second challenge [37]. Overall, this indicates that hemocytes do not remain constantly activated after the initial challenge, but rather mount a stronger response to a second ookinete midgut invasion in the presence of the gut microbiota (Fig 1B). This is in agreement with the observation that hemocyte mRNAs (TEP1, LRIM1) associated with the midgut went back to basal levels 7 days after the first infection, before the second challenge, but reach reached very high levels 24 hours after the second challenge, indicative of enhanced hemocyte recruitment to the midgut surface [37].

## Can immune memory impact vector-borne disease transmission?

Despite its remarkable plasticity, activation of vector immunity often restricts infection under tolerable levels, instead of completely eliminating the pathogen [52]. Nevertheless, refractory or quasi-refractory populations are observed worldwide [53], and experimental infections of field-caught mosquitoes reveal remarkable variability between individuals. While stochastic variations [54] and genetic components [55,56] are important factors driving this heterogeneity, differences in individual life histories also shape host immunological status [57–60].

Immune priming, immune tolerance, and immune training are likely to be important modulators of individual vector competence. Recently, features of trained immunity were described in nonimmune vertebrate cells, such as epithelial and mesenchymal cells [61], and the role of the gut microbiota has been proposed [62]. Interestingly, the vector gut microbiota is also a major driver of mosquito immunity [63]. It remains to be established whether insect immune priming and trained immunity share some regulatory signaling pathways, such as those activated by the gut microbiota.

Thus, priming the immune system of insect vectors is a potential strategy to reduce disease transmission. Manipulation of larvae would be an interesting strategy to reduce vectorial capacity, as infection or immune challenge of larvae, nutritional manipulation, and intraspecific competition have been shown to enhance immunity in adult stages [26,27,64] and logistics to target larval breeding sites are well established [65–67]. While the epigenetic regulation of immunometabolism is an important factor driving susceptibility in vertebrates (and a target for therapeutics), it has only recently started to be investigated in models of disease vectors [40,68,69]. Further identification of molecular markers will make it possible to establish the frequency and intensity of such events in natural populations.
